# Correction: Gogusev et al. Idiopathic Abdominal Wall Endometrioma: Case Report with Investigation of the Pathological, Molecular Cytogenetic and Cell Growth Features In Vitro. *Int. J. Mol. Sci.* 2025, *26*, 775

**DOI:** 10.3390/ijms27073312

**Published:** 2026-04-07

**Authors:** Jean Gogusev, Yves Lepelletier, Henri Cohen, Olivier Ami, Pierre Validire

**Affiliations:** 1 Institut Cochin, INSERM UMR 1016, CNRS 8104, Université Paris Cité, 22 Rue Méchain, 75014 Paris, France; 2 W-MedPhys, 128 Rue la Boétie, 75008 Paris, France; yves.lepelletier@u-paris.fr; 3 Institut Imagine, Université Paris Cité, 24 Boulevard Montparnasse, 75015 Paris, France; 4 INSERM UMR 1163, Laboratory of Cellular and Molecular Basis of Normal Hematopoiesis and Hematological Disorders: Therapeutical Implications, 24 Boulevard Montparnasse, 75015 Paris, France; 5 Service de Chirurgie Gynécologique, Institut Mutualiste Montsouris, 42 Bd Jourdan, 75014 Paris, France; 6 Ramsay Générale de Santé, Clinique de la Muette, 46-48 rue Nicolo, 75116 Paris, France; 7 Service d’Anatomie Pathologique, Institut Mutualiste Montsouris, 42 Bd Jourdan, 75014 Paris, France

In the original publication [1], there was a mistake in the reference citation published. An unintentional error occurred in Reference 32 as this paper has since been retracted. Thus, this reference has been deleted and replaced by another one. However, the retracted reference citation does not affect the results and conclusions of this research. 

We have replaced this reference in the citation list, namely,

32. Jiang, Y.; Jahagirdar, B.N.; Reinhardt, R.L.; Schwartz, R.E.; Keene, C.D.; Ortiz-Gonzalez, X.R.; Reyes, M.; Lenvik, T.; Lund, T.; Blackstad, M.; et al. Pluripotency of mesenchymal stem cells derived from adult marrow. *Nature* **2002**, *418*, 41–49. https://doi.org/10.1038/nature00870.

with

32. Gargett, C.E.; Schwab, K.E.; Zillwood, R.M.; Nguyen, H.P.T.; Wu, D. Isolation and culture of epithelial progenitors and mesenchymal stem cells from human endometrium. *Biol. Reprod.* **2009**, *80*, 1136–1145. https://doi.org/10.1095/biolreprod.108.075226.

**Text Correction**

There was an error in the original publication. In Section 4.1, “tp” has been changed to “to” in “Additional imaging studies were performed tp distinguish for differential diagnosis with keloid.”

The authors state that the scientific conclusions are unaffected. This correction was approved by the Academic Editor. The original publication has also been updated.
